# 2-Amino-4-(4-bromo­phen­yl)-8-trifluoro­methyl-3,4-dihydropyrimido[1,2-*a*][1,3,5]triazin-6(5*H*)-one^1^

**DOI:** 10.1107/S1600536809007612

**Published:** 2009-03-06

**Authors:** Anton V. Dolzhenko, Nikhil Sachdeva, Geok Kheng Tan, Lip Lin Koh, Wai Keung Chui

**Affiliations:** a Department of Pharmacy, Faculty of Science, National University of Singapore, 18 Science Drive 4, Singapore 117543, Singapore; b Department of Chemistry, Faculty of Science, National University of Singapore, 3 Science Drive 3, Singapore 117543, Singapore

## Abstract

The title compound, C_13_H_9_BrF_3_N_5_O, crystallizes with two independent mol­ecules in the asymmetric unit. The pyrimidine rings of the mol­ecules are planar [maximum deviations 0.053 (3) and 0.012 (3) Å], while the triazine rings adopt flattened half-boat conformations with the *p*-bromo­phenyl rings in the flagpole positions. The crystal packing is stabilized by a three-dimensional network of inter­molecular N—H⋯N, N—H⋯O and N—H⋯F hydrogen bonds.

## Related literature

For the crystal structure of 7,7-dimethyl-2-phenyl-6,7-di­hydro-1,2,4-triazolo[1,5-*a*][1,3,5]triazin-5-amine, see: Dolzhenko *et al.* (2007 ▶). For the preparation of benzo-fused analogues, see: Dolzhenko *et al.* (2008*a* ▶). For the previous report in this series, see: Dolzhenko *et al.* (2008*b* ▶).
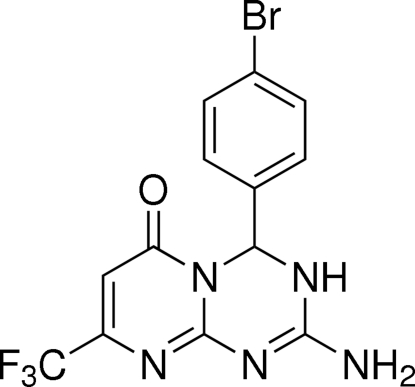


## Experimental

### 

#### Crystal data

C_13_H_9_BrF_3_N_5_O
*M* *_r_* = 388.16
Orthorhombic, 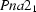

*a* = 10.0531 (4) Å
*b* = 29.9108 (13) Å
*c* = 10.1945 (4) Å
*V* = 3065.4 (2) Å^3^
*Z* = 8
Mo *K*α radiation
μ = 2.73 mm^−1^
*T* = 223 K
0.46 × 0.34 × 0.20 mm

#### Data collection

Bruker SMART APEX CCD diffractometer
Absorption correction: multi-scan (*SADABS*; Sheldrick, 2001 ▶) *T*_min_ = 0.367, *T*_max_ = 0.612 (expected range = 0.348–0.580)
20728 measured reflections
6287 independent reflections
4979 reflections with *I* > 2σ(*I*)
*R*_int_ = 0.037

#### Refinement

*R*[*F* ^2^ > 2σ(*F* ^2^)] = 0.052
*wR*(*F* ^2^) = 0.138
*S* = 1.04
6287 reflections
432 parameters
16 restraints
H atoms treated by a mixture of independent and constrained refinement
Δρ_max_ = 1.50 e Å^−3^
Δρ_min_ = −0.74 e Å^−3^
Absolute structure: Flack (1983 ▶), 2044 Friedel pairs
Flack parameter: 0.011 (10)

### 

Data collection: *SMART* (Bruker, 2001 ▶); cell refinement: *SAINT* (Bruker, 2001 ▶); data reduction: *SAINT*; program(s) used to solve structure: *SHELXS97* (Sheldrick, 2008 ▶); program(s) used to refine structure: *SHELXL97* (Sheldrick, 2008 ▶); molecular graphics: *SHELXTL* (Sheldrick, 2008 ▶); software used to prepare material for publication: *SHELXTL*.

## Supplementary Material

Crystal structure: contains datablocks I, New_Global_Publ_Block. DOI: 10.1107/S1600536809007612/ng2543sup1.cif

Structure factors: contains datablocks I. DOI: 10.1107/S1600536809007612/ng2543Isup2.hkl

Additional supplementary materials:  crystallographic information; 3D view; checkCIF report

## Figures and Tables

**Table 1:** Hydrogen-bond geometry (Å, °)

*D*—H⋯*A*	*D*—H	H⋯*A*	*D*⋯*A*	*D*—H⋯*A*
N1—H1*N*⋯N2^i^	0.87 (2)	2.15 (2)	3.005 (5)	171 (5)
N5—H5*A*⋯O2^ii^	0.895 (14)	2.09 (3)	2.905 (5)	152 (5)
N5—H5*B*⋯N4^i^	0.892 (14)	2.25 (2)	3.095 (6)	159 (5)
N5—H5*B*⋯F1^i^	0.892 (14)	2.46 (4)	3.054 (5)	124 (4)
N6—H6*N*⋯N7^iii^	0.902 (19)	2.10 (3)	2.967 (5)	160 (5)
N10—H10*A*⋯O1	0.89 (2)	2.03 (3)	2.885 (5)	162 (5)
N10—H10*B*⋯N9^iii^	0.90 (2)	2.15 (2)	3.041 (5)	171 (5)

Symmetry codes: (i) 
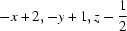
; (ii) 
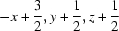
; (iii) 
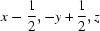
.

**Fig. 1.**
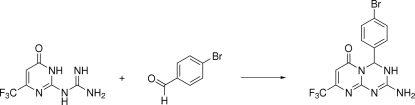
The synthesis of 2-amino-4-(4-bromophenyl)-8-trifluoromethyl-3,4-dihydro-6H-pyrimido[1,2-a][1,3,5]triazin-6-one

**Fig. 2.**
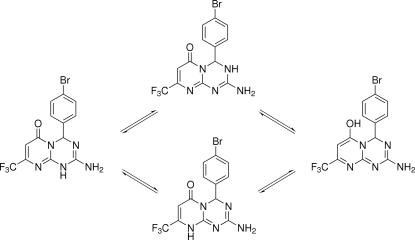
Tautomerism in the title compound

**Fig. 3.**
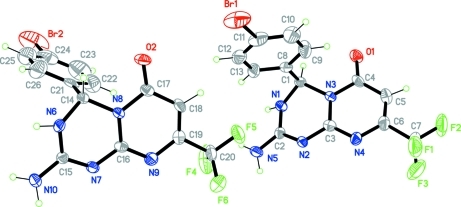
The molecular structure of 2-amino-4-(4-bromophenyl)-8-trifluoromethyl-3,4-dihydro-6H-pyrimido[1,2-a][1,3,5]triazin-6-one with the atomic numbering scheme. Displacement ellipsoids are drawn at the 50% probability level.

**Fig. 4.**
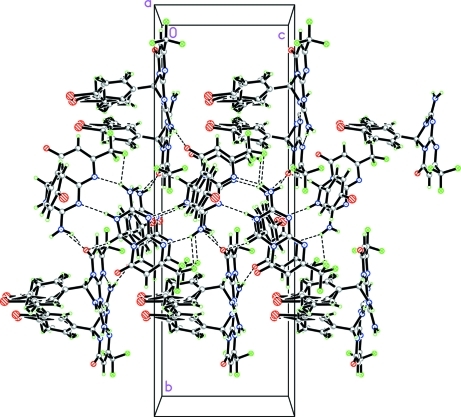
Molecular parking in the crystal, viewed along the a axis. Hydrogen bonds are shown as dashed lines.

## supplementary crystallographic information

### Comment

The title compound was synthesized *via* thermal cyclocondensation of 6-oxo-4-trifluoromethyl-1,6-dihydropyrimidin-2-yl guanidine with *p*-bromobenzaldehyde (Fig.1) using the methodology that we successfully applied previously for the preparation of its benzofused analogues (Dolzhenko *et al.*, 2008*a*). In general, the synthesized compound might be involved in tautomerism with four possible tautomers (Fig. 2). However, only one tautomeric form *viz.* 2-amino-4-(4-bromophenyl)-8-trifluoromethyl-3,4-dihydro-6*H*-pyrimido[1,2-*a*][1,3,5]triazin-6-one was found in the crystal.

The compound crystallized with two independent molecules (A and B) in the asymmetric unit (Fig. 3). The pyrimidine rings in the molecules are planar with maximum deviations 0.0525 (26) Å (C3) and 0.0118 (32) Å (C18) from the planes C3/N3/C4—C6/N4 and C16/N8/C17—C19/N9 of molecules A and B, respectively. Similarly to structurally related 7,7-dimethyl-2-phenyl-6,7-dyhydro-1,2,4-triazolo[1,5-*a*][1,3,5]triazin-5-amine (Dolzhenko *et al.*, 2007), the triazine rings in the molecules adopt flattened half-boat conformations with atoms N2 and N7 at the sterns and *sp^3^*-hybridized atoms C1 and C14 at the bows with *p*-bromophenyl rings as flagpoles. However, the molecules are significantly different in the geometry at bridgehead nitrogen atoms (N3 and N8) and *sp^3^*-hybridized atoms C1 and C14 of the triazine rings.The torsion angles C4—N3—C1—N1 and C17—N8—C14—N6 are 136.7 (4)° and 150.4 (3)°, respectively. In molecule B, the bond N8—C14 is located almost in the phenyl ring (C21—C26) plane: the torsion angle N8—C14—C21—C26 is 2.6 (5)°. The corresponding torsion angle N3—C1—C8—C9 of molecule A is 42.5 (6)°. The N1—C2, N2—C2, N5—C2 bond distances in A and N6—C15, N7—C15, N10—C15 in B are similar that suggests guanidine-like electron delocalization in N1/N2/N5/C2 and N6/N7/N10/C15 fragments of the molecules.

The crystal packing is stabilized by three-dimensional network of intramolecular N—H···N, N—H···O and N—H···F hydrogen bonds (Table 1, Fig. 4).

### Experimental

2-Amino-4-(4-bromophenyl)-8-trifluoromethyl-3,4-dihydro-6*H*-pyrimido[1,2-*a*][1,3,5]triazin-6-one was synthesized from 6-oxo-4-trifluoromethyl-1,6-dihydropyrimidin-2-yl guanidine and *p*-bromobenzaldehyde according to general method reported by Dolzhenko *et al.* (2008*a*). Single crystals suitable for crystallographic analysis were grown by slow evaporation of the solution in ethyl acetate / methanol.

### Refinement

N-bound H atoms were located in a difference map and refined freely. C-bound H atoms were positioned geometrically (C—H = 0.94 or 0.99 Å) and were constrained in a riding motion approximation with *U*_iso_(H) = 1.2*U*_eq_(C). One of the BrC_6_H_4_ parts is disordered into two parts at 89:11 ratio.

### Crystal data

**Table:**

C_13_H_9_BrF_3_N_5_O	*D*_x_ = 1.682 Mg m^−^^3^
*M**_r_* = 388.16	Melting point: 516 K
Orthorhombic, *P**n**a*2_1_	Mo *K*α radiation, λ = 0.71073 Å
Hall symbol: P 2c -2n	Cell parameters from 5069 reflections
*a* = 10.0531 (4) Å	θ = 2.4–25.1°
*b* = 29.9108 (13) Å	µ = 2.73 mm^−^^1^
*c* = 10.1945 (4) Å	*T* = 223 K
*V* = 3065.4 (2) Å^3^	Block, colourless
*Z* = 8	0.46 × 0.34 × 0.20 mm
*F*(000) = 1536	

### Data collection

**Table:**

Bruker SMART APEX CCD diffractometer	6287 independent reflections
Radiation source: fine-focus sealed tube	4979 reflections with *I* > 2σ(*I*)
graphite	*R*_int_ = 0.037
φ and ω scans	θ_max_ = 27.5°, θ_min_ = 1.4°
Absorption correction: multi-scan (*SADABS*; Sheldrick, 2001)	*h* = −12→13
*T*_min_ = 0.367, *T*_max_ = 0.612	*k* = −38→38
20728 measured reflections	*l* = −13→11

### Refinement

**Table:**

Refinement on *F*^2^	Secondary atom site location: difference Fourier map
Least-squares matrix: full	Hydrogen site location: inferred from neighbouring sites
*R*[*F*^2^ > 2σ(*F*^2^)] = 0.052	H atoms treated by a mixture of independent and constrained refinement
*wR*(*F*^2^) = 0.138	*w* = 1/[σ^2^(*F*_o_^2^) + (0.0646*P*)^2^ + 2.1384*P*] where *P* = (*F*_o_^2^ + 2*F*_c_^2^)/3
*S* = 1.04	(Δ/σ)_max_ = 0.001
6287 reflections	Δρ_max_ = 1.50 e Å^−^^3^
432 parameters	Δρ_min_ = −0.73 e Å^−^^3^
16 restraints	Absolute structure: Flack (1983), 2044 Friedel pairs
Primary atom site location: structure-invariant direct methods	Flack parameter: 0.011 (10)

### Special details

**Table:**

Geometry. All e.s.d.'s (except the e.s.d. in the dihedral angle between two l.s. planes) are estimated using the full covariance matrix. The cell e.s.d.'s are taken into account individually in the estimation of e.s.d.'s in distances, angles and torsion angles; correlations between e.s.d.'s in cell parameters are only used when they are defined by crystal symmetry. An approximate (isotropic) treatment of cell e.s.d.'s is used for estimating e.s.d.'s involving l.s. planes.
Refinement. Refinement of *F*^2^ against ALL reflections. The weighted *R*-factor *wR* and goodness of fit *S* are based on *F*^2^, conventional *R*-factors *R* are based on *F*, with *F* set to zero for negative *F*^2^. The threshold expression of *F*^2^ > σ(*F*^2^) is used only for calculating *R*-factors(gt) *etc*. and is not relevant to the choice of reflections for refinement. *R*-factors based on *F*^2^ are statistically about twice as large as those based on *F*, and *R*- factors based on ALL data will be even larger.

### Fractional atomic coordinates and isotropic or equivalent isotropic displacement parameters (Å^2^)

**Table:**

	*x*	*y*	*z*	*U*_iso_*/*U*_eq_	Occ. (<1)
O1	1.0316 (3)	0.34878 (10)	1.2353 (3)	0.0346 (7)	
F1	0.9317 (4)	0.36022 (11)	1.7842 (4)	0.0634 (10)	
F2	0.8409 (4)	0.30866 (11)	1.6709 (4)	0.0750 (12)	
F3	0.7371 (4)	0.37004 (16)	1.7065 (5)	0.0873 (14)	
N1	1.0194 (3)	0.47967 (11)	1.2317 (4)	0.0259 (7)	
H1N	1.012 (5)	0.4927 (15)	1.156 (3)	0.031*	
N2	0.9826 (3)	0.48272 (10)	1.4583 (3)	0.0237 (7)	
N3	1.0181 (3)	0.41352 (10)	1.3521 (3)	0.0232 (7)	
N4	0.9342 (4)	0.41721 (11)	1.5665 (4)	0.0290 (7)	
N5	0.9597 (4)	0.54572 (11)	1.3299 (4)	0.0335 (8)	
H5A	0.919 (5)	0.5568 (15)	1.401 (3)	0.040*	
H5B	0.982 (5)	0.5632 (14)	1.262 (4)	0.040*	
C1	1.0910 (4)	0.43826 (13)	1.2510 (4)	0.0250 (8)	
H1A	1.0904	0.4209	1.1683	0.030*	
C2	0.9892 (4)	0.50251 (13)	1.3412 (4)	0.0249 (8)	
C3	0.9789 (4)	0.43766 (13)	1.4607 (4)	0.0258 (8)	
C4	0.9958 (4)	0.36750 (13)	1.3367 (5)	0.0288 (9)	
C5	0.9359 (5)	0.34747 (15)	1.4486 (5)	0.0387 (10)	
H5	0.9122	0.3171	1.4478	0.046*	
C6	0.9137 (5)	0.37273 (15)	1.5558 (5)	0.0358 (10)	
C7	0.8558 (7)	0.35237 (18)	1.6796 (6)	0.0542 (15)	
C8	1.2338 (4)	0.44558 (14)	1.2947 (5)	0.0279 (9)	
C9	1.3053 (5)	0.41197 (16)	1.3543 (6)	0.0454 (13)	
H9	1.2654	0.3838	1.3664	0.054*	
C10	1.4348 (5)	0.41866 (18)	1.3968 (6)	0.0519 (14)	
H10	1.4826	0.3954	1.4372	0.062*	
C11	1.4917 (4)	0.45984 (18)	1.3788 (6)	0.0441 (12)	
C12	1.4249 (5)	0.49351 (18)	1.3178 (6)	0.0450 (12)	
H12	1.4660	0.5214	1.3044	0.054*	
C13	1.2959 (5)	0.48627 (15)	1.2758 (5)	0.0362 (10)	
H13	1.2495	0.5095	1.2336	0.043*	
Br1	1.66612 (4)	0.47071 (3)	1.44122 (8)	0.0697 (2)	
O2	0.7222 (3)	0.09824 (9)	0.9889 (3)	0.0338 (7)	
F4	1.2813 (4)	0.10516 (13)	1.0040 (5)	0.0782 (13)	
F5	1.1837 (3)	0.05015 (8)	1.0941 (4)	0.0578 (10)	
F6	1.2446 (3)	0.10589 (11)	1.2081 (5)	0.0631 (10)	
N6	0.7243 (3)	0.22885 (11)	1.0553 (4)	0.0271 (8)	
H6N	0.653 (3)	0.2463 (13)	1.073 (5)	0.032*	
N7	0.9576 (3)	0.22901 (10)	1.0690 (4)	0.0261 (7)	
N8	0.8395 (3)	0.16152 (10)	1.0287 (4)	0.0237 (7)	
N9	1.0675 (3)	0.16188 (11)	1.0790 (4)	0.0288 (8)	
N10	0.8364 (4)	0.29270 (11)	1.1091 (4)	0.0319 (8)	
H10A	0.909 (3)	0.3050 (15)	1.144 (5)	0.038*	
H10B	0.762 (3)	0.3090 (15)	1.104 (6)	0.038*	
C20	1.1928 (5)	0.09342 (14)	1.0926 (6)	0.0377 (11)	
C14	0.7273 (4)	0.18807 (12)	0.9806 (5)	0.0258 (9)	
H14	0.6443	0.1713	0.9985	0.031*	
C15	0.8407 (4)	0.25037 (12)	1.0759 (4)	0.0231 (8)	
C16	0.9555 (4)	0.18357 (12)	1.0582 (4)	0.0246 (8)	
C17	0.8279 (4)	0.11509 (13)	1.0201 (4)	0.0255 (8)	
C18	0.9505 (4)	0.09218 (13)	1.0451 (5)	0.0287 (9)	
H18	0.9540	0.0608	1.0441	0.034*	
C19	1.0602 (4)	0.11631 (13)	1.0699 (4)	0.0291 (9)	
Br2	0.74894 (14)	0.22662 (3)	0.38715 (10)	0.0924 (5)	0.899 (3)
C21	0.7359 (4)	0.19636 (19)	0.8349 (3)	0.0312 (13)	0.899 (3)
C22	0.6379 (4)	0.22284 (19)	0.7782 (4)	0.0475 (14)	0.899 (3)
H22	0.5695	0.2348	0.8304	0.057*	0.899 (3)
C23	0.6409 (4)	0.23160 (16)	0.6443 (4)	0.0599 (19)	0.899 (3)
H23	0.5747	0.2495	0.6060	0.072*	0.899 (3)
C24	0.7420 (4)	0.21389 (14)	0.5671 (3)	0.0509 (16)	0.899 (3)
C25	0.8400 (4)	0.18741 (14)	0.6238 (3)	0.0621 (19)	0.899 (3)
H25	0.9084	0.1754	0.5716	0.075*	0.899 (3)
C26	0.8370 (4)	0.17865 (16)	0.7577 (3)	0.0463 (16)	0.899 (3)
H26	0.9033	0.1607	0.7960	0.056*	0.899 (3)
Br3	0.8608 (7)	0.21715 (19)	0.3838 (6)	0.054 (2)*	0.101 (3)
C21A	0.756 (6)	0.197 (3)	0.829 (2)	0.045*	0.101 (3)
C22A	0.668 (5)	0.219 (3)	0.747 (3)	0.062*	0.101 (3)
H22A	0.5867	0.2298	0.7806	0.075*	0.101 (3)
C23A	0.699 (4)	0.225 (2)	0.616 (2)	0.062*	0.101 (3)
H23A	0.6392	0.2402	0.5601	0.075*	0.101 (3)
C24A	0.818 (3)	0.2090 (17)	0.5660 (15)	0.062*	0.101 (3)
C25A	0.907 (4)	0.1867 (17)	0.6480 (18)	0.062*	0.101 (3)
H25A	0.9876	0.1758	0.6145	0.075*	0.101 (3)
C26A	0.875 (5)	0.181 (2)	0.7796 (19)	0.062*	0.101 (3)
H26A	0.9351	0.1654	0.8350	0.075*	0.101 (3)

### Atomic displacement parameters (Å^2^)

**Table:**

	*U*^11^	*U*^22^	*U*^33^	*U*^12^	*U*^13^	*U*^23^
O1	0.0404 (17)	0.0260 (14)	0.0375 (18)	−0.0012 (12)	−0.0011 (14)	−0.0105 (13)
F1	0.103 (3)	0.0442 (17)	0.0428 (19)	−0.0128 (17)	0.0068 (19)	0.0091 (14)
F2	0.119 (3)	0.0427 (17)	0.063 (2)	−0.039 (2)	0.021 (2)	0.0051 (17)
F3	0.071 (3)	0.101 (3)	0.090 (3)	0.004 (2)	0.044 (2)	0.032 (3)
N1	0.0288 (17)	0.0241 (16)	0.0247 (18)	0.0042 (13)	−0.0028 (14)	−0.0010 (14)
N2	0.0241 (15)	0.0214 (15)	0.0255 (19)	0.0018 (12)	0.0019 (14)	0.0007 (14)
N3	0.0235 (16)	0.0197 (14)	0.0265 (19)	0.0033 (12)	−0.0003 (13)	−0.0010 (13)
N4	0.0333 (18)	0.0281 (16)	0.0258 (18)	−0.0022 (14)	0.0025 (15)	0.0023 (15)
N5	0.047 (2)	0.0229 (17)	0.031 (2)	0.0074 (15)	0.0066 (18)	0.0020 (16)
C1	0.0256 (19)	0.0257 (19)	0.024 (2)	0.0051 (15)	0.0029 (15)	−0.0009 (16)
C2	0.0203 (18)	0.0254 (18)	0.029 (2)	0.0001 (14)	−0.0008 (16)	0.0024 (16)
C3	0.0210 (17)	0.0277 (19)	0.029 (2)	0.0016 (14)	−0.0037 (16)	−0.0034 (17)
C4	0.027 (2)	0.0196 (18)	0.040 (2)	0.0005 (15)	−0.0060 (18)	−0.0055 (18)
C5	0.047 (2)	0.028 (2)	0.041 (3)	−0.0088 (18)	−0.003 (2)	0.002 (2)
C6	0.034 (2)	0.032 (2)	0.041 (3)	−0.0076 (18)	0.000 (2)	0.005 (2)
C7	0.071 (4)	0.041 (3)	0.050 (4)	−0.013 (3)	0.016 (3)	0.005 (3)
C8	0.027 (2)	0.031 (2)	0.026 (2)	0.0038 (16)	0.0029 (17)	−0.0034 (17)
C9	0.034 (2)	0.037 (2)	0.065 (4)	−0.0027 (19)	−0.006 (2)	0.019 (2)
C10	0.035 (2)	0.059 (3)	0.062 (4)	0.012 (2)	−0.008 (2)	0.018 (3)
C11	0.020 (2)	0.067 (3)	0.045 (3)	0.000 (2)	−0.009 (2)	−0.015 (3)
C12	0.033 (2)	0.044 (3)	0.058 (3)	−0.009 (2)	0.006 (2)	−0.012 (2)
C13	0.029 (2)	0.033 (2)	0.047 (3)	−0.0071 (18)	−0.001 (2)	0.000 (2)
Br1	0.0269 (2)	0.1078 (5)	0.0743 (4)	0.0028 (3)	−0.0111 (3)	−0.0396 (4)
O2	0.0328 (16)	0.0275 (14)	0.0410 (19)	−0.0115 (12)	−0.0019 (13)	−0.0060 (13)
F4	0.054 (2)	0.075 (2)	0.105 (3)	0.0289 (17)	0.040 (2)	0.035 (2)
F5	0.0513 (18)	0.0253 (13)	0.097 (3)	0.0075 (12)	−0.0090 (18)	−0.0024 (16)
F6	0.0541 (19)	0.0514 (17)	0.084 (3)	0.0087 (15)	−0.0318 (19)	−0.0024 (19)
N6	0.0182 (16)	0.0241 (16)	0.039 (2)	0.0027 (12)	−0.0038 (14)	−0.0013 (15)
N7	0.0238 (16)	0.0228 (15)	0.0317 (19)	−0.0017 (12)	−0.0012 (14)	−0.0055 (15)
N8	0.0236 (16)	0.0170 (14)	0.0306 (19)	−0.0037 (13)	0.0000 (13)	−0.0022 (13)
N9	0.0234 (16)	0.0271 (16)	0.036 (2)	−0.0004 (13)	−0.0010 (15)	−0.0021 (16)
N10	0.0282 (18)	0.0223 (17)	0.045 (2)	−0.0005 (13)	−0.0071 (16)	−0.0066 (16)
C20	0.036 (2)	0.024 (2)	0.053 (3)	0.0041 (17)	0.004 (2)	0.006 (2)
C14	0.0235 (19)	0.0188 (18)	0.035 (2)	−0.0034 (14)	−0.0021 (16)	0.0006 (16)
C15	0.0263 (19)	0.0203 (17)	0.0227 (19)	0.0001 (14)	−0.0019 (16)	0.0032 (16)
C16	0.0223 (18)	0.0272 (19)	0.024 (2)	−0.0066 (15)	0.0011 (16)	−0.0003 (16)
C17	0.033 (2)	0.0238 (18)	0.0199 (19)	−0.0006 (16)	0.0032 (16)	−0.0034 (16)
C18	0.032 (2)	0.0179 (17)	0.036 (2)	−0.0016 (16)	−0.0008 (18)	0.0034 (17)
C19	0.031 (2)	0.028 (2)	0.028 (2)	0.0018 (16)	0.0023 (18)	0.0035 (18)
Br2	0.1629 (13)	0.0842 (6)	0.0300 (3)	−0.0488 (7)	−0.0104 (5)	0.0130 (4)
C21	0.044 (3)	0.019 (2)	0.030 (3)	−0.017 (2)	−0.012 (2)	−0.0023 (19)
C22	0.053 (4)	0.051 (4)	0.039 (3)	0.004 (3)	−0.011 (3)	0.008 (3)
C23	0.084 (5)	0.051 (4)	0.045 (4)	−0.008 (4)	−0.024 (4)	0.012 (3)
C24	0.087 (5)	0.043 (3)	0.023 (3)	−0.027 (3)	−0.007 (3)	0.000 (3)
C25	0.092 (6)	0.063 (4)	0.031 (3)	−0.010 (4)	0.006 (3)	−0.011 (3)
C26	0.065 (4)	0.046 (3)	0.028 (3)	0.001 (3)	−0.003 (3)	−0.004 (2)

### Geometric parameters (Å, °)

**Table:**

O1—C4	1.229 (5)	N6—H6N	0.902 (19)
F1—C7	1.331 (8)	N7—C15	1.339 (5)
F2—C7	1.319 (6)	N7—C16	1.364 (5)
F3—C7	1.334 (7)	N8—C16	1.373 (5)
N1—C2	1.344 (6)	N8—C17	1.396 (5)
N1—C1	1.446 (5)	N8—C14	1.464 (5)
N1—H1N	0.87 (2)	N9—C16	1.317 (5)
N2—C2	1.334 (6)	N9—C19	1.368 (5)
N2—C3	1.348 (5)	N10—C15	1.311 (5)
N3—C3	1.379 (5)	N10—H10A	0.89 (2)
N3—C4	1.403 (5)	N10—H10B	0.90 (2)
N3—C1	1.465 (5)	C20—C19	1.516 (6)
N4—C3	1.318 (6)	C14—C21	1.509 (5)
N4—C6	1.351 (6)	C14—C21A	1.591 (9)
N5—C2	1.331 (5)	C14—H14	0.9900
N5—H5A	0.895 (14)	C17—C18	1.433 (6)
N5—H5B	0.892 (14)	C18—C19	1.343 (6)
C1—C8	1.519 (6)	C18—H18	0.9400
C1—H1A	0.9900	Br2—C24	1.875 (3)
C4—C5	1.423 (7)	C21—C22	1.3900
C5—C6	1.347 (8)	C21—C26	1.3900
C5—H5	0.9400	C22—C23	1.3900
C6—C7	1.517 (7)	C22—H22	0.9400
C8—C9	1.377 (6)	C23—C24	1.3900
C8—C13	1.381 (6)	C23—H23	0.9400
C9—C10	1.387 (7)	C24—C25	1.3900
C9—H9	0.9400	C25—C26	1.3900
C10—C11	1.371 (8)	C25—H25	0.9400
C10—H10	0.9400	C26—H26	0.9400
C11—C12	1.361 (8)	Br3—C24A	1.921 (7)
C11—Br1	1.893 (4)	C21A—C22A	1.3900
C12—C13	1.383 (7)	C21A—C26A	1.3900
C12—H12	0.9400	C22A—C23A	1.3900
C13—H13	0.9400	C22A—H22A	0.9400
O2—C17	1.219 (5)	C23A—C24A	1.3900
F4—C20	1.316 (6)	C23A—H23A	0.9400
F5—C20	1.298 (5)	C24A—C25A	1.3900
F6—C20	1.341 (7)	C25A—C26A	1.3900
N6—C15	1.352 (5)	C25A—H25A	0.9400
N6—C14	1.439 (5)	C26A—H26A	0.9400
			
C2—N1—C1	115.8 (4)	F5—C20—F4	108.7 (4)
C2—N1—H1N	119 (3)	F5—C20—F6	107.1 (4)
C1—N1—H1N	123 (3)	F4—C20—F6	105.4 (4)
C2—N2—C3	117.5 (3)	F5—C20—C19	113.0 (4)
C3—N3—C4	123.9 (4)	F4—C20—C19	111.7 (4)
C3—N3—C1	116.3 (3)	F6—C20—C19	110.5 (4)
C4—N3—C1	119.7 (3)	N6—C14—N8	107.3 (3)
C3—N4—C6	116.3 (4)	N6—C14—C21	112.5 (4)
C2—N5—H5A	113 (3)	N8—C14—C21	112.0 (4)
C2—N5—H5B	126 (4)	N6—C14—C21A	112 (3)
H5A—N5—H5B	121 (5)	N8—C14—C21A	106 (2)
N1—C1—N3	106.2 (3)	C21—C14—C21A	7(2)
N1—C1—C8	112.7 (3)	N6—C14—H14	108.3
N3—C1—C8	109.8 (3)	N8—C14—H14	108.3
N1—C1—H1A	109.3	C21—C14—H14	108.3
N3—C1—H1A	109.3	C21A—C14—H14	114.4
C8—C1—H1A	109.3	N10—C15—N7	120.2 (3)
N5—C2—N2	119.8 (4)	N10—C15—N6	118.1 (3)
N5—C2—N1	118.1 (4)	N7—C15—N6	121.6 (3)
N2—C2—N1	122.0 (4)	N9—C16—N7	117.7 (3)
N4—C3—N2	119.2 (4)	N9—C16—N8	121.7 (3)
N4—C3—N3	120.7 (3)	N7—C16—N8	120.6 (3)
N2—C3—N3	120.0 (4)	O2—C17—N8	120.0 (4)
O1—C4—N3	119.7 (4)	O2—C17—C18	126.8 (4)
O1—C4—C5	127.3 (4)	N8—C17—C18	113.1 (3)
N3—C4—C5	113.0 (4)	C19—C18—C17	118.9 (4)
C6—C5—C4	119.0 (4)	C19—C18—H18	120.6
C6—C5—H5	120.5	C17—C18—H18	120.6
C4—C5—H5	120.5	C18—C19—N9	126.3 (4)
C5—C6—N4	126.3 (4)	C18—C19—C20	120.6 (4)
C5—C6—C7	120.9 (4)	N9—C19—C20	113.1 (4)
N4—C6—C7	112.8 (4)	C22—C21—C26	120.0
F2—C7—F1	107.1 (5)	C22—C21—C14	117.5 (3)
F2—C7—F3	107.7 (5)	C26—C21—C14	122.5 (3)
F1—C7—F3	106.2 (5)	C21—C22—C23	120.0
F2—C7—C6	112.7 (5)	C21—C22—H22	120.0
F1—C7—C6	112.1 (4)	C23—C22—H22	120.0
F3—C7—C6	110.8 (5)	C24—C23—C22	120.0
C9—C8—C13	118.0 (4)	C24—C23—H23	120.0
C9—C8—C1	121.2 (4)	C22—C23—H23	120.0
C13—C8—C1	120.9 (4)	C23—C24—C25	120.0
C8—C9—C10	121.5 (4)	C23—C24—Br2	120.2 (2)
C8—C9—H9	119.2	C25—C24—Br2	119.7 (2)
C10—C9—H9	119.2	C24—C25—C26	120.0
C11—C10—C9	118.7 (5)	C24—C25—H25	120.0
C11—C10—H10	120.7	C26—C25—H25	120.0
C9—C10—H10	120.7	C25—C26—C21	120.0
C12—C11—C10	121.3 (4)	C25—C26—H26	120.0
C12—C11—Br1	118.9 (4)	C21—C26—H26	120.0
C10—C11—Br1	119.7 (4)	C22A—C21A—C26A	120.0
C11—C12—C13	119.3 (5)	C22A—C21A—C14	123 (3)
C11—C12—H12	120.4	C26A—C21A—C14	117 (3)
C13—C12—H12	120.4	C21A—C22A—C23A	120.0
C8—C13—C12	121.2 (5)	C21A—C22A—H22A	120.0
C8—C13—H13	119.4	C23A—C22A—H22A	120.0
C12—C13—H13	119.4	C24A—C23A—C22A	120.0
C15—N6—C14	117.9 (3)	C24A—C23A—H23A	120.0
C15—N6—H6N	112 (3)	C22A—C23A—H23A	120.0
C14—N6—H6N	128 (3)	C23A—C24A—C25A	120.0
C15—N7—C16	117.8 (3)	C23A—C24A—Br3	119.9 (4)
C16—N8—C17	124.2 (3)	C25A—C24A—Br3	120.0 (4)
C16—N8—C14	117.9 (3)	C26A—C25A—C24A	120.0
C17—N8—C14	117.0 (3)	C26A—C25A—H25A	120.0
C16—N9—C19	115.7 (3)	C24A—C25A—H25A	120.0
C15—N10—H10A	118 (3)	C25A—C26A—C21A	120.0
C15—N10—H10B	122 (3)	C25A—C26A—H26A	120.0
H10A—N10—H10B	119 (5)	C21A—C26A—H26A	120.0
			
C2—N1—C1—N3	49.4 (5)	C14—N6—C15—N10	160.7 (4)
C2—N1—C1—C8	−70.9 (5)	C14—N6—C15—N7	−22.5 (6)
C3—N3—C1—N1	−45.3 (4)	C19—N9—C16—N7	178.7 (4)
C4—N3—C1—N1	136.7 (4)	C19—N9—C16—N8	−0.3 (6)
C3—N3—C1—C8	76.9 (4)	C15—N7—C16—N9	−166.1 (4)
C4—N3—C1—C8	−101.0 (4)	C15—N7—C16—N8	12.9 (6)
C3—N2—C2—N5	163.1 (4)	C17—N8—C16—N9	1.2 (6)
C3—N2—C2—N1	−13.9 (6)	C14—N8—C16—N9	−167.5 (4)
C1—N1—C2—N5	160.7 (4)	C17—N8—C16—N7	−177.8 (4)
C1—N1—C2—N2	−22.3 (6)	C14—N8—C16—N7	13.5 (6)
C6—N4—C3—N2	170.1 (4)	C16—N8—C17—O2	−177.5 (4)
C6—N4—C3—N3	−8.0 (6)	C14—N8—C17—O2	−8.7 (6)
C2—N2—C3—N4	−160.4 (4)	C16—N8—C17—C18	−0.2 (6)
C2—N2—C3—N3	17.8 (5)	C14—N8—C17—C18	168.6 (4)
C4—N3—C3—N4	9.9 (6)	O2—C17—C18—C19	175.5 (4)
C1—N3—C3—N4	−167.9 (4)	N8—C17—C18—C19	−1.6 (6)
C4—N3—C3—N2	−168.2 (3)	C17—C18—C19—N9	2.6 (7)
C1—N3—C3—N2	13.9 (5)	C17—C18—C19—C20	−178.1 (4)
C3—N3—C4—O1	178.5 (4)	C16—N9—C19—C18	−1.6 (7)
C1—N3—C4—O1	−3.8 (6)	C16—N9—C19—C20	179.1 (4)
C3—N3—C4—C5	−3.8 (5)	F5—C20—C19—C18	−4.4 (7)
C1—N3—C4—C5	174.0 (4)	F4—C20—C19—C18	118.5 (5)
O1—C4—C5—C6	174.3 (4)	F6—C20—C19—C18	−124.5 (5)
N3—C4—C5—C6	−3.2 (6)	F5—C20—C19—N9	175.0 (4)
C4—C5—C6—N4	5.0 (8)	F4—C20—C19—N9	−62.1 (6)
C4—C5—C6—C7	−177.2 (5)	F6—C20—C19—N9	54.9 (5)
C3—N4—C6—C5	0.8 (7)	N6—C14—C21—C22	−56.3 (4)
C3—N4—C6—C7	−177.1 (4)	N8—C14—C21—C22	−177.3 (3)
C5—C6—C7—F2	4.5 (8)	C21A—C14—C21—C22	−147 (25)
N4—C6—C7—F2	−177.4 (5)	N6—C14—C21—C26	123.7 (4)
C5—C6—C7—F1	125.4 (5)	N8—C14—C21—C26	2.6 (5)
N4—C6—C7—F1	−56.6 (6)	C21A—C14—C21—C26	33 (24)
C5—C6—C7—F3	−116.3 (6)	C26—C21—C22—C23	0.0
N4—C6—C7—F3	61.8 (6)	C14—C21—C22—C23	180.0 (5)
N1—C1—C8—C9	160.8 (5)	C21—C22—C23—C24	0.0
N3—C1—C8—C9	42.5 (6)	C22—C23—C24—C25	0.0
N1—C1—C8—C13	−19.2 (6)	C22—C23—C24—Br2	−178.6 (3)
N3—C1—C8—C13	−137.5 (4)	C23—C24—C25—C26	0.0
C13—C8—C9—C10	1.3 (8)	Br2—C24—C25—C26	178.6 (3)
C1—C8—C9—C10	−178.7 (5)	C24—C25—C26—C21	0.0
C8—C9—C10—C11	0.1 (9)	C22—C21—C26—C25	0.0
C9—C10—C11—C12	−1.5 (9)	C14—C21—C26—C25	−180.0 (5)
C9—C10—C11—Br1	177.8 (5)	N6—C14—C21A—C22A	−66 (4)
C10—C11—C12—C13	1.4 (9)	N8—C14—C21A—C22A	177 (3)
Br1—C11—C12—C13	−177.9 (4)	C21—C14—C21A—C22A	26 (22)
C9—C8—C13—C12	−1.4 (8)	N6—C14—C21A—C26A	114 (3)
C1—C8—C13—C12	178.6 (5)	N8—C14—C21A—C26A	−3(4)
C11—C12—C13—C8	0.1 (8)	C21—C14—C21A—C26A	−154 (27)
C15—N6—C14—N8	44.6 (5)	C26A—C21A—C22A—C23A	0.0
C15—N6—C14—C21	−79.0 (5)	C14—C21A—C22A—C23A	−180 (6)
C15—N6—C14—C21A	−71 (2)	C21A—C22A—C23A—C24A	0.0
C16—N8—C14—N6	−40.1 (5)	C22A—C23A—C24A—C25A	0.0
C17—N8—C14—N6	150.4 (3)	C22A—C23A—C24A—Br3	179 (4)
C16—N8—C14—C21	83.9 (5)	C23A—C24A—C25A—C26A	0.0
C17—N8—C14—C21	−85.6 (5)	Br3—C24A—C25A—C26A	−179 (4)
C16—N8—C14—C21A	80 (3)	C24A—C25A—C26A—C21A	0.0
C17—N8—C14—C21A	−89 (3)	C22A—C21A—C26A—C25A	0.0
C16—N7—C15—N10	168.1 (4)	C14—C21A—C26A—C25A	180 (6)
C16—N7—C15—N6	−8.7 (6)		

### Hydrogen-bond geometry (Å, °)

**Table:**

*D*—H···*A*	*D*—H	H···*A*	*D*···*A*	*D*—H···*A*
N1—H1N···N2^i^	0.87 (2)	2.15 (2)	3.005 (5)	171 (5)
N5—H5A···O2^ii^	0.90 (1)	2.09 (3)	2.905 (5)	152 (5)
N5—H5B···N4^i^	0.89 (1)	2.25 (2)	3.095 (6)	159 (5)
N5—H5B···F1^i^	0.89 (1)	2.46 (4)	3.054 (5)	124 (4)
N6—H6N···N7^iii^	0.90 (2)	2.10 (3)	2.967 (5)	160 (5)
N10—H10A···O1	0.89 (2)	2.03 (3)	2.885 (5)	162 (5)
N10—H10B···N9^iii^	0.90 (2)	2.15 (2)	3.041 (5)	171 (5)

Symmetry codes: (i) −*x*+2, −*y*+1, *z*−1/2; (ii) −*x*+3/2, *y*+1/2, *z*+1/2; (iii) *x*−1/2, −*y*+1/2, *z*.
